# Quantitative and qualitative analysis of the novel antitumor 1,3,4-oxadiazole derivative (GLB) and its metabolites using HPLC-UV and UPLC-QTOF-MS

**DOI:** 10.1038/srep11906

**Published:** 2015-07-07

**Authors:** Pu Li, Xin Wang, Jian Li, Zhi-Yun Meng, Shu-Chun Li, Zhong-Jun Li, Ying-Yuan Lu, Hong Ren, Ya-Qing Lou, Chuang Lu, Gui-Fang Dou, Guo-Liang Zhang

**Affiliations:** 1 Department of Pharmacology, School of Basic Medical Sciences, Peking University, Beijing, 100191, PR. China; 2 Laboratory of Hematological Pharmacology, Beijing Institute of Transfusion Medicine, Beijing 100850, PR China; 3 Department of Chemical Biology, School of Pharmaceutical Sciences, Peking University, Beijing, 100191, PR. China; 4 Millennium Pharmaceuticals-Takeda, Cambridge, Massachusetts, USA

## Abstract

Fructose-based 3-acetyl-2,3-dihydro-1,3,4-oxadiazole (GLB) is a novel antitumor agent and belongs to glycosylated spiro-heterocyclic oxadiazole scaffold derivative. This research first reported a simple, specific, sensitive and stable high performance liquid chromatography -ultraviolet detector (HPLC-UV) method for the quantitative determination of GLB in plasma. In this method, the chromatographic separation was achieved with a reversed phase C_18_ column. The calibration curve for GLB was linear at 300 nm. The lower limit of quantification was 10 ng/mL. The precision, accuracy and stability of the method were validated adequately. This method was successfully applied to the pharmacokinetic study in rats for detection of GLB after oral administration. Moreover, the structures of parent compound GLB and its two major metabolites M1 and M2 were identified in plasma using an ultra performance liquid chromatography- electrospray ionization-quadrupole-time of flight- mass spectrometry (UPLC-ESI-QTOF-MS) method. Our results indicated that the di-hydroxylation (M1) and hydroxylation (M2) of GLB are the major metabolites. In conclusion, the present study provided valuable information on an analytical method for the determination of GLB and its metabolites in rats, can be used to support further developing of this antitumor agent.

Fructose-based 3-acetyl-2,3-dihydro-1,3,4-oxadiazole (GLB) is a novel antitumor agent and belongs to glycosylated spiro-heterocyclic oxadiazole scaffold derivative^1^ (Fig. 1A). The introduce of fructose group into the pharmacophore of 1,3,4-oxadiazole molecule enhanced the hydrophobicity, and therefore makes GLB easier across various cellular membranes^2^. Compared to the 1^st^ generation compound, 1,3,4-oxadiazole^3^,^4^,^5^,^6^,^7^,^8^,^9^,^10^,^11^,^12^,^13^,^14^, GLB exhibited more potent antitumor activity and higher orally bioavailable in animals. As a thymidine phosphorylase inhibitor^15^,^16^,^17^,^18^,^19^, GLB is shown to reduce the production and secretion of vascular endothelial growth factor (VEGF), suppress the formation of new blood vessels and especially block the tumor angiogenesis, growth and metastasis *in vivo*^20^. Moreover, it has been reported that GLB inhibited matrix metalloproteinases, induced cell cycle arrest, promoted apoptosis and inhibited proliferation in multiple human carcinoma cell lines including uterine cervix cancer cells, pulmonary adenocarcinoma cells, and prostate cancer cells (PC-3M)^21^. Thus further research is needed for GLB as a potential antitumor candidate.

During the research and development of anticancer agent GLB, the pharmacokinetic profile is needed for understanding the absorption, distribution, metabolism and excretion (ADME) of GLB *in vivo*^22^. The high performance liquid chromatographic - ultraviolet detector (HPLC-UV) is a conventional quantitative analysis method^23^,^24^,^25^,^26^. In according to the ultraviolet absorption wavelength, elution retention time and chromatographic peak area, the content of compound can be accurately measured^27^,^28^,^29^,^30^. Recently, the ultra-performance liquid chromatography- electrospray ionization-quadrupole-time-of-flight mass spectrometry (UPLC-ESI-QTOF-MS) has been also used in pharmacokinetics and qualitative metabolite studies^31^,^32^,^33^,^34^,^35^. Based on comparison of the chromatographic retention times and mass spectral patterns (mass-to-charge ratio, m/z) between the parent drug and their product ions, the structures of metabolites can be identified^36^,^37^,^38^,^39^,^40^,^41^,^42^,^43^. Although many methods for evaluating the biological and pharmacological activity of 1,3,4-oxadiazole and its derivatives have been reported^44^,^45^,^46^,^47^, only a few analytical methods on these compounds in biological matrixes^48^,^49^,^50^. There is a need for an analytical method which can quantitative analyze GLB and its possible metabolites from *in vivo* or *in vitro* study.

The purpose of the present study is to establish a simple, specific, sensitive and reliable HPLC-UV method for determination of GLB in rat plasma. To the best of our knowledge, this is the first report of establishing and validating a HPLC-UV method for the quantitative determination of GLB in rats plasma. Moreover, the structures of GLB’s two metabolites in rat plasma were identified using the UPLC-ESI-QTOF-MS method. In the present research, this HPLC method has been fully validated in terms of specificity, sensitivity, precision, accuracy and stability for quantification of GLB, and then it was successfully applied to the pharmacokinetic study of GLB in rats. This work provided valuable information for further understanding the GLB metabolism and disposition *in vivo*.

## Results and Discussion

### Method development and optimization

In the present study, a simple, specific and sensitive HPLC method was developed for determination of GLB in rat plasma, which did not require the use of expensive and complicated mass spectrometry equipment. The result showed that ultraviolet (UV) wavelength at 300 nm produced chromatograms with the highest GLB areas compared with those at 242 nm. Moreover, several compounds were tested as internal standard (IS), including tinidazole (which was one of the azole compounds)^51^, amiodarone hydrochloride (similar to GLB which contains halogen)^52^, estradiol benzoate and megestrol acetate (both have UV absorption at 280 nm). Megestrol acetate was chosen as an IS for its appropriate UV absorption, retention time and extracted recovery.

On the other hand, different compositions of mobile phase, for example, several combinations of % organic solvents (methanol and acetonitrile) and distilled water were tested and compared. It was found that a mixture of acetonitrile and distilled water (65:35, v/v) was the optimal mobile phase, which achieved better resolution, more symmetric peaks and short retention time for analytes. During method development and optimization of the extraction procedure, several different mixtures of organic solvents were examined, including acetonitrile and ethyl acetate^53^. Ethyl acetate had the highest recovery. Therefore, the plasma sample was extracted in ethyl acetate using a simple liquid-liquid extraction. In order to show the method optimization and support their statements, the parameters of HPLC method optimization for determination of GLB including resolution, symmetry, retention time, and extraction efficiency were listed in the Supplementary Data (Appendix Table 2).

### Method validation

#### Specificity

**Figure 1:**
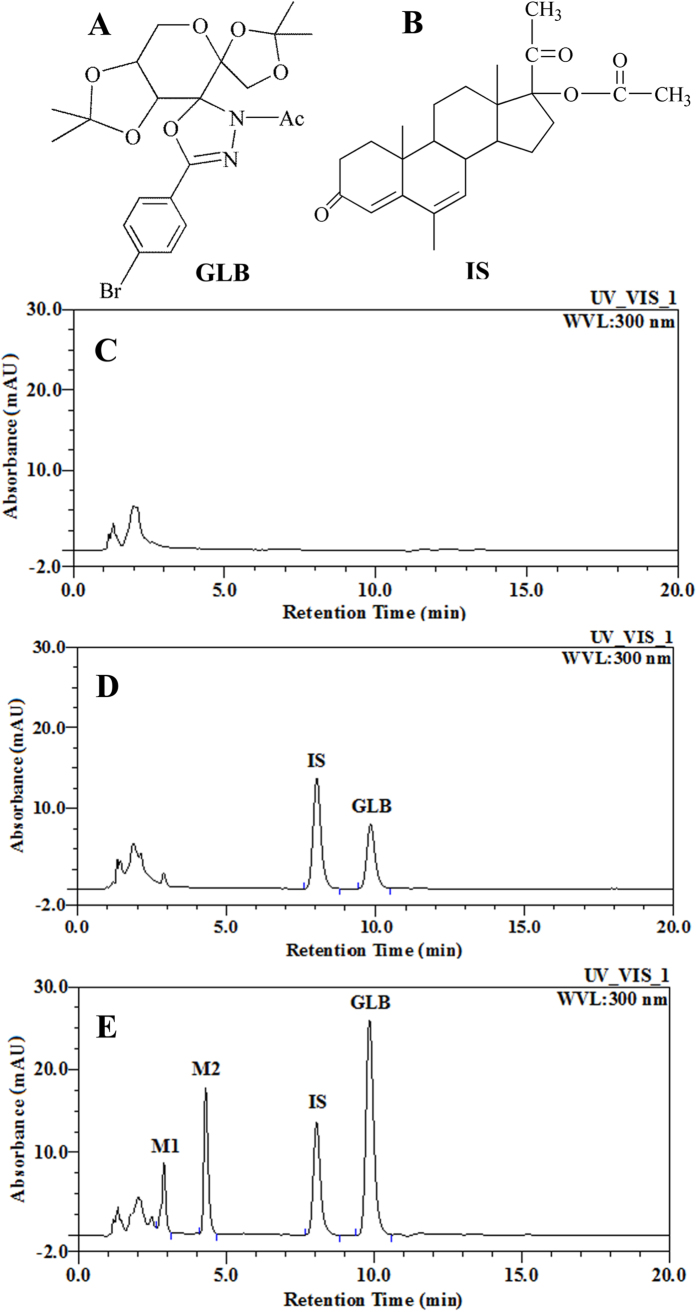
Chemical structures and representative HPLC-UV chromatograms of (**A**) fructose-based 3-acetyl-2,3-dihydro-1,3,4-oxadiazole (GLB); (**B**) megestrol acetate (internal standard, IS); (**C**) rat blank plasma; (**D**) rat blank plasma spiked with fructose-based 3-acetyl-2,3-dihydro- 1,3,4-oxadiazole (GLB, 1 μg/mL) and internal standard (IS, 1 μg/mL) and (**E**) a rat plasma sample at 6 h after a single oral administration of GLB (100 mg/kg, n = 6).

As shown in Fig. 1, specificity was assayed by comparing the chromatograms of rat blank plasma (Fig. 1C), rat blank plasma spiked with GLB and IS (Fig. 1D), and rat plasma collected 6 h after oral administration of GLB, representatively (Fig. 1E). The retention times of GLB and IS was approximately 9.8 and 8.0 min, respectively. In addition, in the plasma HPLC chromatogram, there were two additional chromatographic peaks which have retention times shorter than the parent drug, suggesting possible formation of two polar metabolites^54^. Those peaks were later identified as M1 and M2 in the QTOF analysis. There was no interference from endogenous substances in rat plasma observed in our method.

#### Calibration curve and sensitivity

Calibration curve of GLB showed linearity at the concentration ranging from 0.01 to 8 μg/mL in rat plasma. The average regression equation of the calibration curves can be expressed as y = 0.5530x + 0.0133 (r^2^ = 0.9996, n = 5). The correlation coefficients (r) of the linear regression with a weighing factor of 1/*x*^2^ ranged from 0.9968–0.9998. The lowest limit of quantification (LLOQ) of GLB was 10 ng/mL and the limit of detection (LOD) was 1 ng/mL (S/N > 3) in rat plasma. The lowest concentration on the calibration curve (10 ng/mL) was accepted as LLOQ.

#### Precision, accuracy and recovery

Precision and accuracy were evaluated by assaying five replicates at three concentrations (0.025, 0.5, 4 μg/mL) on the same day (intra-day) and on five different days (inter-day). Accuracy was assessed by the percentage deviation of mean observed from spiked values and expressed as relative error (RE). Precision was expressed as variations value by the relative standard deviation (RSD). As shown in Table 1, the variations of precision values ranged from 2.97% to 7.23% for intra-day and ranged from 2.09% to 7.46% for inter-day, respectively. The accuracy was ranged from 1.24% to 12.1% for intra-day and 1.84% to 3.20% for inter-day respectively. All the values were within the acceptable range.

The recoveries of GLB in plasma samples are showed in Table 1. The plasma samples were extracted by ethyl acetate in a liquid-liquid extraction. The mean extraction recoveries of GLB at the low, medium and high concentrations (0.025, 0.5, 4 μg/mL) were ranged from 97.1% to 108%, achieving an acceptable extraction recovery.

#### Stability

The stability of GLB in rat plasma was determined by analyzing QC samples that was stored in different conditions, including: 1) room-temperature, 2) light irradiation for 24 hours, 3) post-preparation (the extracted samples stored at 4 ^o^C for 48 hours and one week), 4) three cycles of freeze and thaw (freezing at −20 °C for 24 hours and thawing at room temperature), 5) long-term stored in −20 ^o^C for up to 2 weeks, 1 month and 3 months, respectively. All the QC samples for stability assessment were analyzed in triplicate.

Under manifold storage and processing conditions, the stability of GLB in rat plasma was evaluated by analyzing three replicates for QC samples at 0.025, 0.5 and 4 μg/mL. The results summarized in Table 2, showed GLB was stable in plasma storing for 24 hours; 48 hours and 1 week at 4 °C; or 2 weeks, 1 month and 3 months at −20 °C as well as after three freeze-thaw cycles.

### Application of the method to pharmacokinetic study

#### Pharmacokinetics of GLB parent drug

**Figure 2:**
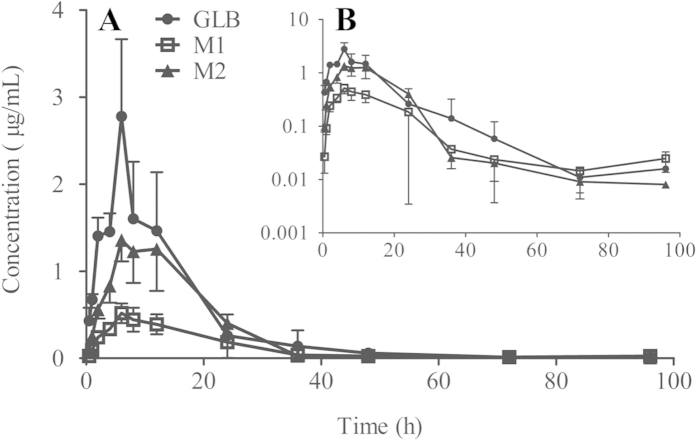
The plasma concentration-time curves (**A**) and semilogdataplot (the y-coordinate was logarithmic coordinates, (**B**) of fructose-based 3-acetyl-2,3-dihydro-1,3,4- oxadiazole (GLB) and its two metabolites M1 and M2 after single oral administration in rat (100 mg/kg, n = 6).

The validated bioanalytical method was applied to the detection of GLB concentration in plasma after a single oral administration of GLB (100 mg/kg, n = 6) in rats. The mean plasma concentration–time curves of GLB, metabolites M1 and M2 are shown in Fig. 2A. At 0.5 h after oral administration, the prototype drug of GLB was detected in rat plasma. The peak concentration of GLB was at 6 h (2.78 ± 0.89 μg/mL) with quantifiable at the 36 h (0.14 ± 0.18 μg/mL). At 96 h time point, low concentration of GLB was still detectable in rat plasma (Fig. 2B). As shown in Table 3, pharmacokinetics of GLB was analyzed by non-compartmental model and the parameters were calculated as follows: C_max_ was 2.78 ± 0.89 μg/mL, T_max_ was 6 h. Moreover, the corresponding values for t_1/2_, AUC_0→96_ and AUC_0-∞_ were 9.24 ± 4.74 h, 33.30 ± 9.10 mg/L·h and 33.49 ± 9.05 mg/L·h respectively. These results suggest that the absorption and elimination of GLB might be slowly in rats and the specific reasons remained to be studied in future experiments.

#### Pharmacokinetics of GLB metabolites

On the other hand, two chromatographic peaks of unknown compounds were found in rat plasma samples (Fig. 1E), which were tentatively named as metabolites M1 (2.8 min) and M2 (4.3 min), both of which had shorter retention time compared to GLB prototype drug; and the areas and heights of the two peaks were highly correlative to the parent GLB concentration - time courses after oral doses. Due to lack of standards of metabolites, the actual concentrations of two metabolites in plasma could not be accurately determined. However, the difference in maximum UV absorption wavelength between metabolites and the parent drug is expected to be not much different, hence, semiquantitative analysis was made to estimate the relative concentrations of metabolites based on the standard curve of GLB. The relative concentration-time curves of two individual metabolites in plasma were shown in Fig. 2. Similar to the parent drug of GLB, both of M1 and M2 could be detected in rat plasma in 0.5 h; furthermore, the values of time to reach the peak concentrations (T_max_) of the two metabolites were also determined to be 6 h suggesting the metabolite formation could be rapid. The concentrations of M1 and M2 were detectable at 96 h time point in rat plasma (See Supplementary Data “Appendix Table 1”). Moreover, the pharmacokinetic parameters of two metabolites M1 and M2 were also evaluated, as well as the results of relative concentrations of metabolites based on the standard curve of GLB (Table 3).

### Identification of GLB and its metabolites in rat plasma

**Figure 3:**
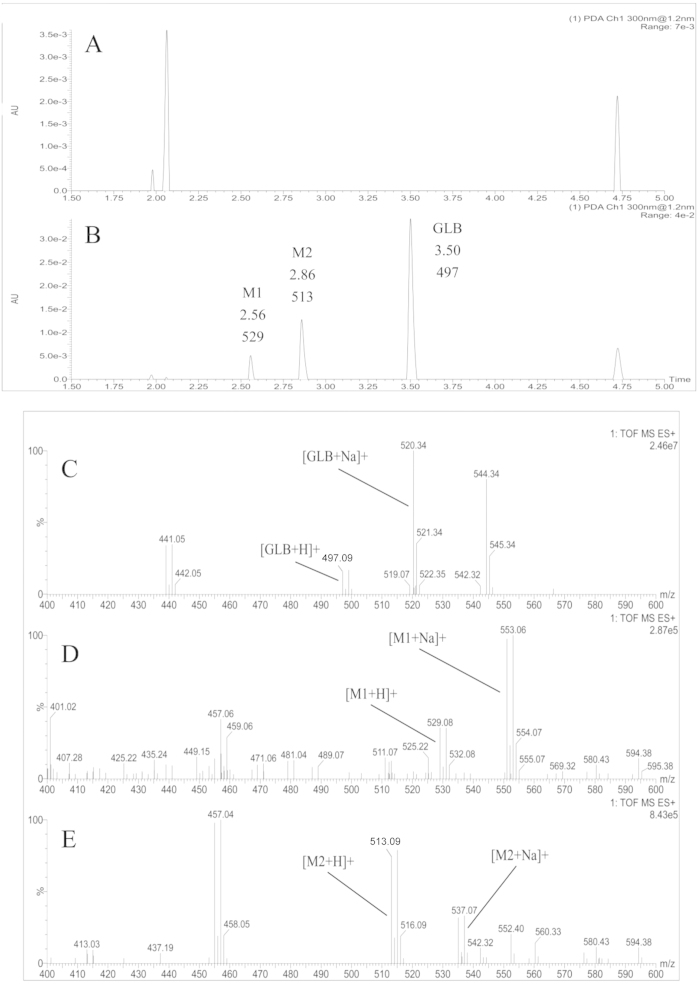
Representative UPLC chromatograms and tandem ESI-QTOF-MS mass spectrograms of (**A**): blank rat plasma; (**B**): GLB (3.50 min and m/z 497.09), metabolite M1 (2.56 min and m/z 529.08) and M2 (2.86 min and m/z 513.09) in a rat plasma sample at 6 h after oral administration GLB (100 mg/kg); (**C**): mass spectrograms of the parent compound GLB; (**D**): metabolites M1 and (**E**): metabolites M2 in positive scan mode using electrospray ionization (ESI) and in-source collisionally induced dissociation detected by UPLC-QTOF-MS/MS in a rat plasma sample at 6 h after a single oral administration of GLB (100 mg/kg).

Because of two unknown chromatographic peaks were detected by HPLC after oral dose GLB in rat plasma, their structures were further identified and the metabolic pathways were investigated using UPLC-MS method. As showed in Table 4, the data of GLB and its major two metabolites from the UPLC ESI-QTOF-MS/MS were compared. Based on the chromatographic retention times and mass spectral patterns (m/z and mass errors) between the parent drug and their product ions, the structures of metabolites of GLB could be estimated, in spite of lacking standards of metabolites. As shown in Fig. 3A,3B, the parent compound GLB profiles at the UPLC elution and mass spectrums were retention time 3.50 min and the molecular ion m/z 497, respectively. Metabolites M1 and M2 were detected at shorter retention times (2.56 and 2.86 min, respectively), and heavier molecular ions as m/z 529 (497 + 32 Da) and 513 (497 + 16 Da), respectively.

**Figure 4:**
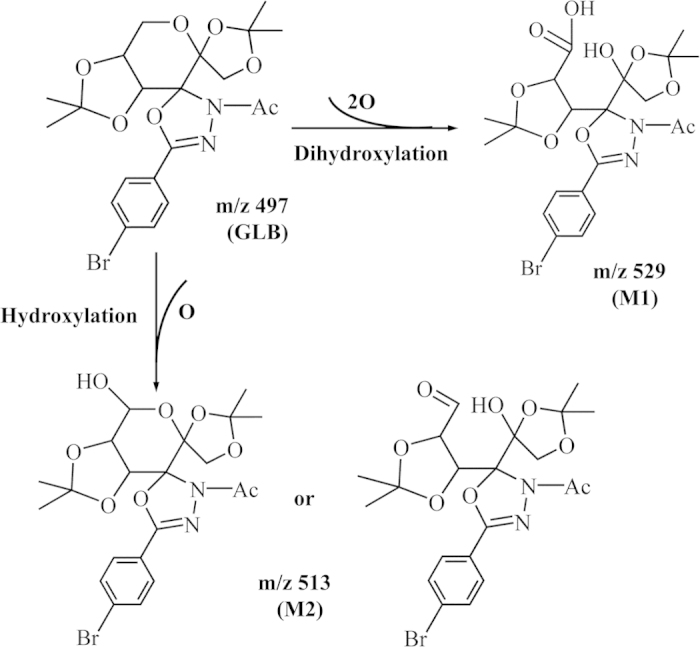


Furthermore, under the tandem mass spectrometry positive scan mode condition, the parent compound GLB was detected as protonated molecular ions at m/z 499 ([M+H]+) and sodium adduct (520, [M+Na]+), and fragment ion m/z 441, respectively (Fig. 3C). The metabolite M1 was detected as protonated molecular ions at m/z 529 ([M+H]+) and sodium adduct (m/z 553, [M+Na]+), and fragment ions m/z 457, 449, 435 and 401, respectively. These molecular ions were 32–34 Da (2O or 2OH) higher compared to the parent compound, may suggest modification of adding two hydroxyl groups into the molecule (Fig. 3D). Therefore, M1 was tentatively identified as the di-hydroxylated metabolite and its possible metabolic pathway might be the di-hydroxylating reaction in phase I metabolism. On the other hand, the metabolite M2 produced protonated molecular ions at m/z 515 ([M+H]+), m/z 537 ([M+Na]+), and fragment ions at m/z 457, 437 and 413, respectively. The molecular ions were 16–17 Da (O or OH) higher than the parent compound (Fig. 3E), suggesting modification might be added one hydroxyl group into the parent molecule. Therefore, M2 could be tentatively identified as a hydroxylated metabolite. These results indicated that fragmentation pathway of GLB, M1 and M2 to support the metabolic site location, suggesting that di-hydroxylation (M1) and hydroxylation (M2) metabolisms might be the major metabolic pathways in rats. Furthermore, the present results showed that both of M1 and M2 had fragment ions at m/z 457, which might be attributed to the hydroxylation of the fructose group fraction (Fig. 4).

## Conclusion

This research is the first report of a simple, specific, sensitive and stable HPLC-UV method for the quantitative determination of GLB in plasma. This method was successfully applied to the pharmacokinetic study of GLB after oral administration in rats. Moreover, the structures of GLB and its two metabolites M1 and M2 were identified by UPLC-ESI-QTOF-MS method suggesting that the di-hydroxylation and hydroxylation of GLB might be its major metabolic pathways in rats. Therefore, the present study provided valuable information on an analytical method for the determination of GLB and its metabolites *in vivo*, can be used to support further developing of this antitumor agent.

## Materials and Methods

### Chemicals and reagents

Fructose-based 3-acetyl-2,3-dihydro-1,3,4-oxadiazole (GLB, Fig. 1A) (purity >98%) was supplied by the Department of Chemical Biology and Pharmaceutics, School of Pharmaceutical Sciences, Beijing University (Beijing, China). Megestrol acetate (internal standard, IS, Fig. 1B) (purity >99%) was acquired from National Institute for Food and Drug Control (Beijing, China). Acetonitrile and methanol were purchased from Fisher Scientific (HPLC grade, Hampton, NH, USA). Extraction agent ethyl acetate was obtained from Merck KGaA (HPLC grade, Darmstadt, Germany).

### Instruments and working conditions

#### High performance liquid chromatography (HPLC)

HPLC-UV analyses were performed with a DIONEX Ultimate 3000 HPLC system (Thermo Fisher Scientific, MA, USA), which included a double-ternary pump (DGP-3600SD), a temperature controlled automatic sampler (WPS-3000SL), a column temperature box (TCC-3000SD), and a ultraviolet (UV) detector (VWD-3400). The UV detection wavelength was set at 300 nm. The chromatographic separation was achieved on a reversed-phase Alltima C18 analytical column (150 mm × 4.6 mm; 5 μm), and the mobile phase was the mixture of acetonitrile and distilled water (65:35, v/v), running at the flow rate of 1.0 mL/min. The temperature of column box was kept at 25 ^o^C. The injection volume was 20 μL. The HPLC data was processed using Chromeleon^TM^ 6.8 software (Thermo Fisher Scientific, MA, USA).

### Ultra-performance liquid chromatography- electrospray ionization- quadrupole -time-of-flight mass spectrometry (UPLC-ESI-QTOF-MS)

The structure identifications of GLB and its metabolites were analyzed using a validated ultra-performance liquid chromatography- electrospray ionization-quadrupole-time-of-flight mass spectrometry (UPLC-ESI-QTOF-MS) method, as described in previous publications^55^,^56^.

Chromatographic experiments were performed on an ACQUITY UPLC system (Waters Corp., Manchester, UK), which equipped with a Waters ACQUITY photodiode array detector (PDA) together with a quaternary pump, an auto-sample injector, an on-line degasser and an automatic thermostatic column oven. UPLC separation was achieved on an ACQUITY UPLC BEH C18 column (100 × 2.1 mm, 1.7 μm, Waters Corp., Milford, MA, USA). The column temperature set at 45 ^o^C. The mobile phase consisted of (A) water containing 0.1% formic acid and (B) acetonitrile containing 0.1% formic acid using a gradient elution of 1 ~ 5% B at 0 ~ 0.5 min, 5 ~ 50% B at 0.5 ~2 min, 50 ~ 80% B at 2 ~ 4.5 min, 80 ~ 5% B at 4.5 ~ 5.5 min. The flow rate was 0.4 mL/min, wavelength was 300 nm, and injection volume was 10 μL.

An ACQUITY Synapt mass spectrometer (Waters Corp., Manchester, UK) was connected to the UPLC system via an electrospray ionization (ESI) interface. Ionization was performed in the positive electrospray (ESI) mode. The mass range was set at *m/z* 100 ~ 1000 Da with a 0.1 s scan time. The conditions used for the ESI source were as follows: capillary voltage, 3.00 kV; sampling cone, 40 V; extraction cone, 3.0 V; source temperature, 100 ^o^C; and desolvation temperature, 280 ^o^C. Nitrogen was used as core gas and desolvation with the flow rate of 50 and 800 L/h, respectively. MS^E^ (where E represents collision energy) analysis was performed on ESI-QTOF/MS setup with collision energy ramp of 6 ~ 30 eV (MS^E^ parameters: low energy, 6 eV; and high energy, 20 ~ 30 eV). The data was acquired and processed using MassLynx^TM^ (version 4.1) software with MS^E^ program (Waters Corp., Manchester, UK).

### Preparation of standards, calibration standard and quality control (QC) samples

Standard stock solutions of GLB and internal standard (IS) (both 512 μg/mL) were prepared by accurately weighing and dissolving the compounds in methanol in 100 mL volumetric flasks at 4 ^o^C. The stock solution of GLB was serially diluted with methanol to provide working solutions (0.16, 0.4, 0.8, 1.6, 8, 16, 64 and 128 μg/mL). The IS working solution (16 μg/mL) was also prepared by diluting stock solution with methanol. Working solution was newly prepared before use.

Calibration standard solutions were prepared by adding 25 μL of working standard solutions of GLB and IS into 375 μL blank rat plasma to achieve final concentrations of GLB of 0.01, 0.025, 0.05, 0.1, 0.5, 1, 4 and 8 μg/mL, and IS concentration of 1 μg/mL, respectively. QC samples were prepared in the same way using the low, medium and high concentrations of 0.025, 0.5 and 4 μg/mL, respectively.

### Validation of HPLC method

The HPLC-UV method was evaluated by specificity, linearity, sensitivity, precision and accuracy, recovery and stability. Specificity was estimated by analyzing blank plasma samples from five different sources to ensure that the retention times of the GLB and IS had no interferences. The linearity of the calibration curves (ranging from 0.01 to 8 μg/mL) was assessed based on five sets of independently prepared calibration curves using weighted (1/*x*^*2*^) least - squares linear regression. Calibration curves had to have correlation coefficients (*r*^*2*^) of 0.99 or better. The sensitivity was expressed by the lower limit of quantification (LLOQ) that could be determined at which both precision and accuracy were less than 20%.

The intra- and inter-day precision and accuracy were determined by analyzing five replicates of QC samples (low-, medium-, and high-concentration of 0.025, 0.5 and 4 μg/mL at each QC level) on the same day and on five consecutive days. The precision was expressed as the relative standard deviation (RSD) that was set to be less than 15% as an acceptance criteria. The accuracy was expressed as relative error (RE), no more than ±15% for the high and medium QCs, and 20% for the low QC. The recovery of GLB from the blank plasma was determined by three QC samples (0.025, 0.5 and 4 μg/mL). Absolute recoveries were calculated by the ratio of the peak areas of the extracted samples vs. the unextracted samples at the same nominal concentration.

### Animal handling and plasma samples collection

Sprague-Dawley rats (Male, 210 ± 20 g, 7–8 weeks) were provided by the Department of Experimental Animal, Beijing University (Beijing, China). Environmental controls for the animal room were set at 22 ± 3 ^o^C with 50 ± 20% relative humidity. This animal study was performed in strict accordance with the recommendations in the Guide for the Care and Use of Laboratory Animals of China Association for Laboratory Animal Science. All animal care and experimental protocols were approved by the Animal Care Committee of Peking University Health Science Center. All sacrifice was performed under pentobarbitone anesthesia, and every effort was made to minimize suffering.

Rats were fasted 12 h prior to the study and were allowed access to food 4 h after oral dosing. Eighteen rats were divided randomly into six groups and rats were sampled at interval in each group. The blood samples at each time point were collected from six rats. The rats were dosed with a suspension of GLB (100 mg/kg, in 0.5% sodium carboxymethyl cellulose CMC-Na solution) and the blood samples were collected at the predose, 0.5, 1, 2, 4, 6, 8, 12, 24, 36, 48, 72 and 96 h post-dosing. The blood samples were centrifuged at 2800 rpm (1473 × g) for 20 min and the plasma was stored at −20 ^o^C until analyzed.

### Sample preparation

The 400 μL plasma sample was transferred into a glass tube, followed by the addition of 25 μL of IS and 2 mL of extraction solvent ethyl acetate. The mixture was vortexed for 3 min to extract GLB and IS from plasma sample and then centrifuged at 2800 rpm (1473 × g) for 20 min. The upper organic phase was transferred into a glass tube. The sample was extracted twice with 2 mL of ethyl acetate for liquid-liquid extraction. The combined upper organic phases were evaporated to dryness at 37 ^o^C under a gentle stream of nitrogen gas. The residue was reconstituted with 100 μL of methanol and was injected 20 μL into the HPLC system. In addition, in order to perform mass spectrometry analyses, the residue was reconstituted with 150 μL of methanol-water (v/v, 1:1), and then centrifuged at 14000 rpm (17968 × g) for 15 min, the upper liquid was transferred into sample vials and 10 μL was injected for UPLC-QTOF/MS analysis.

### Pharmacokinetic parameters and statistic analysis

The pharmacokinetic parameters of GLB were calculated by a non-compartmental pharmacokinetic analysis method using Drug and Statistical Version 3.0 (DAS 3.0) software (the Mathematical Pharmacology Committee, Chinese Pharmacological Society, China). The maximum peak concentration of the drug in plasma (C_max_) and the time to reach the maximum concentration (T_max_) were estimated from the experimental data. The area under the plasma concentration-time curves from 0 to infinity (i.e., AUC_0-∞_) and from 0 to 96 h (AUC_0-96_) were calculated by the trapezoidal summation. The terminal elimination rate constant (*K*e) was derived from the slope of the linear regression curve by fitting the natural logarithms of the terminal concentrations versus time. The terminal elimination half-life (t_1/2_) was calculated by 0.693/*K*e. The data from the quality control (QC) samples and all calculations were calculated using Microsoft Excel 2007 (Microsoft Co., USA). All values were expressed as mean ± SD (Standard Deviation).

## Additional Information

**How to cite this article**: Li, P. *et al.* Quantitative and qualitative analysis of the novel antitumor 1,3,4-oxadiazole derivative (GLB) and its metabolites using HPLC-UV and UPLC-QTOF-MS. *Sci. Rep.* **5**, 11906; doi: 10.1038/srep11906 (2015).

## Supplementary Material

Supplementary Information

## Figures and Tables

**Table 1:** Precision, accuracy and recovery data of fructose-based 3-acetyl-2,3-dihydro- 1,3,4-oxadiazole (GLB) detected by high performance liquid chromatography (HPLC) method in rat plasma (Mean ± SD, n = 5).

**Actual concentration (μg/mL)**	**Measured concentration (μg/mL)**	**Precision (RSD, %)**	**Accuracy (RE, %)**	**Recovery (%)**
Intra-day				
0.025	0.028 ± 0.002	7.23	12.1	101
0.5	0.506 ± 0.019	3.73	1.24	96.5
4	4.055 ± 0.120	2.97	1.38	97.4
Inter-day				
0.025	0.026 ± 0.002	7.46	3.20	108
0.5	0.509 ± 0.011	2.09	1.84	97.1
4	4.108 ± 0.129	3.14	2.70	99.8

RSD: relative standard deviation (%); RE: relative error (%).

**Table 2:** Stability data of fructose-based 3-acetyl-2,3-dihydro-1,3,4-oxadiazole (GLB) detected by high performance liquid chromatography (HPLC) method in rat plasma (Mean ± SD, n = 3).

**Stability**	**Actual concentration (μg/mL)**	**Measured concentration (μg/mL)**	**Precision (RSD, %)**	**Accuracy (RE, %)**
Light and room temperature for 24 hours	0.025	0.026 ± 0.002	7.52	4.17
	0.5	0.486 ± 0.027	5.53	−2.79
	4	3.922 ± 0.055	1.41	−1.94
Post-preparative stability (at 4 °C for 48 hours)	0.025	0.026 ± 0.001	3.63	5.31
	0.5	0.528±0.007	1.25	5.62
	4	4.233 ± 0.196	4.64	5.83
Post-preparative stability (at 4 °C for 1 week)	0.025	0.026 ± 0.001	4.19	3.83
	0.5	0.428 ± 0.010	2.25	−14.4
	4	3.668 ± 0.219	5.98	−8.30
Freeze-thawing 3 cycles (at −20 °C)	0.025	0.025 ± 0.001	−0.75	2.82
	0.5	0.467 ± 0.036	−6.51	7.65
	4	3.732 ± 0.227	−6.71	6.08
Freezing storage stability (at −20 °C for 2 weeks)	0.025	0.027 ± 0.001	4.09	8.17
	0.5	0.535 ± 0.009	1.68	7.10
	4	4.393 ± 0.090	2.04	9.84
Freezing storage stability (at −20 °C for 1 month)	0.025	0.027 ± 0.002	8.29	8.66
	0.5	0.490 ± 0.015	3.01	1.17
	4	4.097 ± 0.213	5.19	−2.27
Freezing storage stability (at −20 °C for 3 months)	0.025	0.023 ± 0.002	−7.12	8.04
	0.5	0.487 ± 0.003	−2.66	−2.04
	4	3.918 ± 0.063	0.71	1.62

**Table 3:** Pharmacokinetic parameters of fructose-based 3-acetyl-2,3-dihydro-1,3,4-oxadiazole (GLB) and its two metabolites M1 and M2 after a single oral administration of GLB (100 mg/kg) in rats (Mean ± SD, n = 6).

**Pharmacokinetic parameters**	**Unit**	**GLB**	**M1**	**M2**
C_max_	mg/L	2.78 ± 0.89	0.54 ± 0.10*	1.56 ± 0.27*
T_max_	h	6 ± 0	7.33 ± 2.73*	8.67 ± 2.73*
AUC_(0-96)_	mg/L·h	33.30 ± 9.10	9.66 ± 3.38*	24.56 ± 6.83
AUC_(0-∞)_	mg/L·h	33.49 ± 9.05	10.05 ± 3.34*	24.68 ± 6.85
t_1/2_	h	9.24 ± 4.74	11.30 ± 5.94	6.82 ± 2.63

Compared with GLB parent drug group, *p < 0.05.

**Table 4:** Retention times and mass spectral patterns (mass-to-charge ratio, m/z) of the compound GLB and its metabolites (M1 and M2) identified by UPLC-QTOF-MS/MS method in rat plasma after oral administration (100 mg/kg).

**Peak**	**Mass Error (ppm)**	**Retention Time (min)**	**Calculated Mass**	**Identification**	**Formula**	**Molecular Ion (m/z)**	**Fragment Ions (m/z)**
GLB	1.0	3.50	496.08	Parent compound	C_21_H_25_N_2_O_7_Br	497 [M+H]+	—
					—	520 [M+Na]+	—
					C_18_H_10_N_2_ O_6_Br	—	441
M1	−0.8	2.56	528.07	Di-hydroxylation	C_21_H_25_N_2_O_9_Br	529 [M+H]+	—
					—	553 [M+Na]+	—
					C_18_H_20_N_2_O_7_Br	—	457
					C_21_H_25_N_2_O_9_	—	449
					C_18_H_18_N_2_O_6_Br	—	435
					C_16_H_18_N_2_O_6_Br	—	401
M2	−0.1	2.86	512.08	Hydroxylation	C_21_H_25_N_2_O_8_Br	513 [M+H]+	—
					—	537 [M+Na]+	—
					C_18_H_20_N_2_O_7_Br	—	457
					C_19_H_20_N_2_O_5_Br	—	437
					C_17_H_20_NO_6_Br	—	413
