# Supplementary material for: Quantitative and qualitative analysis of the novel antitumor 1,3,4-oxadiazole derivative (GLB) and its metabolites using HPLC-UV and UPLC-QTOF-MS
Source: Sci Rep. 2015 Jul 7;5:11906. doi: 10.1038/srep11906 (PMC4493701; doi:10.1038/srep11906)
Supplement: Supplementary Information [file srep11906-s1.pdf]

Quantitative and qualitative analysis of the novel antitumor 1,3,4-oxadiazole derivative (GLB) and its metabolites using HPLC-UV and UPLC-QTOF-MS

Pu Li [1]*, Xin Wang [1]*, Jian Li [2], Zhi-Yun Meng [2], Shu-Chun Li [3], Zhong-Jun Li [3], Ying-Yuan Lu [1], Hong Ren [1], Ya-Qing Lou [1], Chuang Lu [4], Gui-Fang Dou [2] & Guo-Liang Zhang [1]

Supplementary data

Appendix Table 1. Plasma concentration - time courses of fructose-based 3-acetyl-2,3-dihydro-1,3,4-oxadiazole (GLB) and its two metabolites M1 and M2 after a single oral dose of 100 mg/kg in rat plasma (Mean ± SD, n= 6).

Time point （h）	Plasma concentration (μg/mL）		
	GLB	M1	M2
0.5	0.043±0.151	0.027±0.014	0.098±0.020
1	0.673±0.065	0.091±0.021	0.250±0.049
2	1.405±0.210	0.242±0.055	0.558±0.101
4	1.455±0.210	0.336±0.059	0.833±0.193
6	2.779±0.887	0.517±0.113	1.357±0.244
8	1.602±0.656	0.443±0.137	1.228±0.361
12	1.466±0.672	0.390±0.114	1.254±0.480
24	0.262±0.241	0.185±0.182	0.402±0.447
36	0.139±0.183	0.037±0.015	0.026±0.010
48	0.058±0.063	0.024±0.020	0.020±0.011
72	0.011±0.005	0.015±0.009	0.010±0.005
96	0.016±0.017	0.025±0.011	0.010±0.000

Appendix Table 2. The parameters of HPLC method optimization and validation for determination of fructose-based 3-acetyl-2,3-dihydro-1,3,4-oxadiazole (GLB) and megestrol acetate (internal standard, IS), including resolution, symmetry, retention time, and extraction efficiency in rat plasma.

Parameter	GLB	IS
Retention time (min)	9.8	8.0
Resolution	3.6	3.6
Symmetry	1.26	1.27
Extraction efficiency (%)	102	106
Theoretical plate number	6288	5234

Appendix Figure Legend.

Appendix Figure 1. Full wavelength ultraviolet scanning of fructose-based 3-acetyl-2,3-dihydro-1,3,4-oxadiazole (GLB).

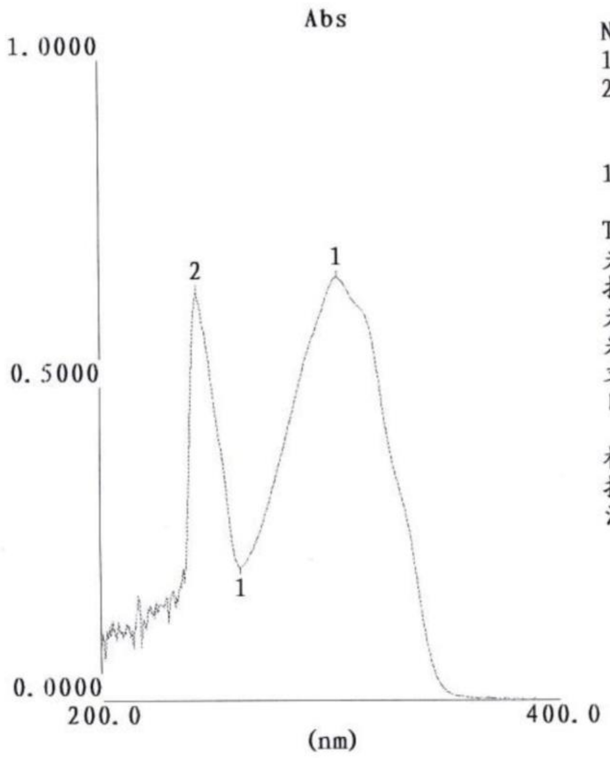

---峰---

No.	nm	Abs
1	303.50	0.646
2	242.00	0.624

---谷---

1	261.00	0.201

TU-1901
光度模式: Abs
扫描速度: 快速
光谱带宽: 2 nm
采样间隔: 0.50 nm
文件名:
日期: 2010-4-30 10: 33

样品: GLB
操作者:
注释:
